# Correction Approach for Delta Function Convolution Model Fitting of Fluorescence Decay Data in the Case of a Monoexponential Reference Fluorophore

**DOI:** 10.1007/s10895-015-1583-4

**Published:** 2015-06-11

**Authors:** Clifford B. Talbot, João Lagarto, Sean Warren, Mark A. A. Neil, Paul M. W. French, Chris Dunsby

**Affiliations:** Photonics Group, Department of Physics, Imperial College London, Prince Consort Road, London, SW7 2AZ UK; Centre for Histopathology, Imperial College London, Du Cane Road, London, W12 0NN UK

**Keywords:** Fluorescence lifetime, Time-resolved spectroscopy, Delta function convolution method (DFCM), Fluorescence decay curve fitting, Reference reconvolution

## Abstract

A correction is proposed to the Delta function convolution method (DFCM) for fitting a multiexponential decay model to time-resolved fluorescence decay data using a monoexponential reference fluorophore. A theoretical analysis of the discretised DFCM multiexponential decay function shows the presence an extra exponential decay term with the same lifetime as the reference fluorophore that we denote as the residual reference component. This extra decay component arises as a result of the discretised convolution of one of the two terms in the modified model function required by the DFCM. The effect of the residual reference component becomes more pronounced when the fluorescence lifetime of the reference is longer than all of the individual components of the specimen under inspection and when the temporal sampling interval is not negligible compared to the quantity (τ_R_^−1^ – τ^−1^)^−1^, where τ_R_ and τ are the fluorescence lifetimes of the reference and the specimen respectively. It is shown that the unwanted residual reference component results in systematic errors when fitting simulated data and that these errors are not present when the proposed correction is applied. The correction is also verified using real data obtained from experiment.

## Introduction

Fluorescence lifetime measurements have a range of photophysical, biological and biomedical applications. The fluorescence lifetime can report on the local environment or state of the fluorophore and it can be used to distinguish between particular fluorescent species. Within the field of biomedical optics, the fluorescence lifetime can report on, for example, protein-protein interactions in FRET, metabolic rate, the disease state of tissue matrix components, and provide many other molecular readouts, resulting in its increasing application in clinical and biology laboratory settings [1, 2]. Fluorescence lifetime data from most biological specimens, however, present complex fluorescent decay profiles and robust methods to analyse such data are required to fully exploit the potential of fluorescence lifetime measurements.

Fluorescence lifetime measurements are carried out in either the time-domain or the frequency domain [1]. For each approach the analysis of lifetime data must take account of the instrument response in order to recover an accurate representation of the specimen fluorescence decay profile. This paper concerns time domain fluorescence lifetime measurements that are analysed by fitting the experimental data to a model of the fluorescence decay. For most experimental systems, the temporal instrument response function (IRF), which is the apparent signal that is measured for an ideal Dirac Delta function input signal, typically presents a FWHM on the order of a few 100’s of picoseconds and its shape is often complex with sub-structure that distorts the measured fluorescence decay profiles of specimens under inspection. This is of particular importance when the specimen exhibits a multi-exponential fluorescence decay, since the impact of the IRF on the measured signal (which is the convolution of the actual fluorescence signal with the IRF) can distort the apparent contributions of the individual decay components to the recovered fluorescence profile. Thus the shape of the IRF can significantly affect lifetime measurements, even when only the mean lifetime is required, or when a single exponential decay model is fitted to a complex decay profile. It is therefore important to incorporate a measurement of the IRF in the analysis of the fluorescence decay profile – otherwise lifetime measurements cannot be compared between different instruments. Typically in the curve fitting procedure, the fluorescence decay model (such as a multi-exponential decay) is first convolved with the IRF measurement before comparison with the measured fluorescence decay profile of the specimen under inspection.

Since the shape and/or position of the IRF can change when adjusting instrumentation settings such as PMT gain, electronic detection thresholds, laser repetition rate or laser diode drive current, it is usually necessary to measure the IRF for each set of fluorescence lifetime measurements. Furthermore, some detectors exhibit a spectrally dependent temporal response, known as the “colour effect”, due to the velocity of the initial photoelectron [1, 3--5] and so the IRF needs to be determined for each spectral channel of a fluorescence lifetime measurement. Ideally this would be undertaken using a calibration sample that provides a near instantaneous response across the spectral range of the detector. In general, however, it is difficult to find such a fluorescent calibration sample although, for multiphoton excitation, it is possible to utilise the plasmon enhanced luminescence from gold nanorods, which provide a broadband ultrafast (i.e. sub-picosecond) emission that we have previously demonstrated to be a convenient calibration sample for IRF measurements in a multispectral multiphoton microscope [5]. Unfortunately an equivalent calibration sample providing broadband ultrafast emission for single photon excitation in the visible has not yet been identified and, in practice, temporal IRF are often measured using an ultrashort excitation pulse scattered from the sample plane to provide an “impulse response”. Unfortunately this does not provide the IRF for the spectral region of the detected fluorescence emission and scattering specimens often cause unwanted artefacts caused by multiple reflections of the excitation pulses within the instrument. Furthermore, such a temporal IRF measurement at the excitation wavelength may require removal of the laser blocking filter as well as a modification of any configuration in front of the detector providing spectral resolution and this can be time-consuming and impractical on a regular basis, especially for real-world instrumentation, e.g. applied to clinical or industrial applications.

For single photon excited lifetime measurements, an IRF in the desired spectral window can sometime be approximated using a calibration specimen based on a fluorophore with a very short fluorescence lifetime. However, the fluorophores typically used [6--9] are dyes with lifetimes on the order of tens of picoseconds, which is significant compared to the resolution of time-resolved detection systems such as those based on time-correlated single photon counting (TCSPC) and so the measured IRF is not an impulse response. The impact of the non-zero lifetime of the calibration specimen can be approximately compensated by shifting the measured IRF profile in time before or during the fitting procedure. However, this becomes less effective when analysing the fluorescence decay profiles of specimens with short fluorescence lifetimes [10]. Furthermore, the selection of dyes available with very short lifetime and useable quantum yield is limited; the emission spectra of the dyes proposed by Luchowski et al. and Szabelski et al. [6--9] only extend upwards from ~525 nm and do not cover the spectral range of the autofluorescent compounds found in tissue [11] or genetically expressed fluorescent proteins in the blue spectral region [12].

An alternative approach to directly measuring the IRF is to make a reference measurement using a calibration fluorophore with known decay characteristics, for example, a single exponential decay profile with a known lifetime [3, 13--19]. The IRF can be extracted from this measurement for subsequent use in the fitting procedure by performing deconvolution of the measured reference fluorophore decay data with the known reference decay profile (e.g. a single exponential decay) [3, 13]. However, the deconvolution step is sensitive to noise on the reference decay measurement and can be bypassed by using the technique of *reference convolution* [14], which is less sensitive to noise [15]. As discussed below, this technique entails mathematically modifying the model of the specimen fluorescence decay such that, when it is convolved with the measured reference fluorescence decay profile, it provides the desired curve for direct comparison with the measured specimen fluorescence decay profile during curve fitting. This is commonly referred to as the Delta function convolution method (DFCM) [17]. The modification of the specimen decay model can be generalised to any reference decay function and so, in theory, this technique does not require a short or single-exponential lifetime reference fluorophore. Thus a much wider range of reference fluorophores is available [20].

The DFCM has been tested through Monte Carlo simulations [21--23] and through its application to fitting experimental data [10, 14, 17, 21, 23]. However, several of these studies have also reported that the performance of the technique decreases under some conditions which have been associated with: longer reference lifetimes [23]; cases where the reference lifetime is shorter than twice the channel width [22]; cases where the reference lifetime is much longer than the IRF [10] or cases when the specimen lifetime(s) are short compared to the reference decay [10, 14, 21--23]. Limitations in the accuracy of the DFCM have previously been qualitatively attributed to: attempting to fit multi-exponential decays where one component is weak [22], noise on the measured reference decay [14, 23] or only using the early portion of the reference decay when fitting short specimen decays [10].

We have found that the DFCM technique fails when analysing data from fluorophores exhibiting fast decays similar to those associated with tissue autofluorescence and this has motivated us to seek a quantitative explanation for the limitations of the DFCM. For the first time, to the best of our knowledge, we show analytically that these limitations are due to a mismatch in the terms input to the DFCM model function when one of the input terms is discretised. We propose a modification to the DFCM to correct for this mismatch and verify our technique using simulated and experimental fluorescence lifetime data.

## Theory

In conventional non-linear least squares fitting of fluorescence decays, the impulse response function, IRF, of the system is convolved with the model fluorescence decay function, *F*_mod_, of the specimen under inspection to yield a calculated fluorescence intensity decay profile, *I*_mod_, as shown in Eq. 1(a). The parameters of *F*_mod_ then are adjusted to obtain the best fit between *I*_mod_ and the measured decay of a specimen under investigation. We will refer to this as “impusle convolution”. In reference convolution (known in the literature as the Delta function convolution method, DFCM), instead of directly recording the impulse response of the system, the fluorescence decay profile of a reference specimen, *R*, is recorded and the physical decay model is modified (to ${\overset{\sim }{F}}_{\mod{}}$) such that the calculated decay profile is given by Eq. 1(b).

1 $$ \begin{array}{l}{I}_{\mod{}} = {F}_{\mod{}}\ast IRF\kern1.5em (a)\hfill \\ {}{I}_{\mathrm{DFCM}}={\tilde{F}}_{\mod{}}\ast R\kern2.25em (b)\hfill \end{array} $$

This is a well-established technique and it has been shown that the fluorescence lifetime of the reference specimen can also be included as a fit parameter and that its decay does not need to be mono-exponential [10, 14, 15, 17, 21, 22]. However, these relaxations of prior knowledge come at the cost of an increased number of fitted parameters, which in turn requires more detected photons to reach a given accuracy. For the purpose of this paper, we will assume that the reference decay is a mono-exponential with a known fluorescence lifetime, *τ*_R_, and amplitude, *a*_R_. We will also make the common assumption that the specimen decay model is a sum of exponential components with lifetimes and amplitudes *τ*_i_ and *a*_i_. In this case the generalised model function used in the DFCM (see [17] for derivation), is shown in Eq. 2(a). By substituting Eq. 2(a) into Eq. 1(b) and defining the combined lifetime, τ_i_′ = (τ_R_^−1^ – τ_i_^−1^)^−1^, the expression for the calculated fluorescence decay profile is obtained, as shown by Eq. 2(b).

2 $$ \begin{array}{l}{\tilde{F}}_{\mod{}}(t)={\displaystyle \sum_i\frac{a_i}{a_R}\left\{\delta (t)+\left(\frac{1}{\tau_R} - \frac{1}{\tau_i}\right)A\left(t,{\tau}_i\right)\right\}\ }\kern1em (a)\hfill \\ {}\hfill {I}_{\mathrm{DFCM}}(t)={\displaystyle \sum_i\frac{a_i}{a_R}\left\{\underset{\mathrm{Term}\ 1}{\underbrace{R(t)}}+\frac{1}{\tau_i^{\prime }}\left(\underset{\mathrm{Term}\ 2}{\underbrace{\left[A\left({t}^{\prime },{\tau}_i\right)\ast R\left({t}^{\prime}\right)\right](t)}}\right)\right\}}\ (b)\hfill \\ {}A\left(t,\tau \right)=\left\{\begin{array}{c}\hfill \exp \left(-\frac{t}{\tau}\right),\ for\ t\ \ge\ 0\hfill \\ {}\hfill 0,\ for\ t<0\hfill \end{array}\right.\kern1.5em (c)\hfill \end{array} $$

Equation 2(b) can be derived using Laplace or Fourier transforms [15, 17] and can be readily verified for the case where the IRF corresponds to a Delta function at *t* = 0, such that the convolution of the IRF with the reference decay function gives *R*(*t*) = *a*_*R*_*A*(*t*,*τ*_*R*_). If this is substituted for *R* in Eq. 2(b), the convolution (term 2 of Eq. 2(b)) becomes an integral with limits set by noting that the fluorescence decay profiles are zero for *t* < 0. The resulting equation is shown below (Eq. 3(a)) and the result of the integration is shown in Eq. 3(b). The second part of term 2 balances with term 1 of 3(b), leaving a term which is equal to the *i*^th^ multi-exponential decay component, *a*_*i*_exp(−*t*/*τ*_*i*_).

3 $$ \begin{array}{l}{I}_{\mathrm{DFCM}\hbox{-} \mathrm{test}}(t)={\displaystyle \sum_i{a}_i\left\{\underset{\mathrm{Term}\ 1}{\underbrace{ \exp \left(-\frac{t}{\tau_R}\right)}}+\underset{\mathrm{Term}\ 2}{\underbrace{\frac{1}{\tau_i^{\prime }}{\displaystyle \underset{0}{\overset{t}{\int }} \exp \left( - \frac{t - t^{\prime }}{\tau_i}\right) \exp \left( - \frac{t^{\prime }}{\tau_R}\right)dt^{\prime }}}}\right\},}\kern0.5em for\ t\ \ge\ 0\kern1.25em \left(\mathrm{a}\right)\hfill \\ {}\begin{array}{ll}{I}_{\mathrm{DFCM}\hbox{-} \mathrm{test}}(t)={\displaystyle \sum_i{a}_i\left\{\underset{\mathrm{Term}\ 1}{\underbrace{ \exp \left(-\frac{t}{\tau_R}\right)}}+\underset{\mathrm{Term}\ 2}{\underbrace{ \exp \left(-\frac{t}{\tau_i}\right) - \exp \left(-\frac{t}{\tau_R}\right)}}\right\}},\hfill & \kern0.5em for\ t\ \ge\ 0\kern1em \left(\mathrm{b}\right)\hfill \end{array}\hfill \end{array} $$

As detailed in the introduction, however, several studies have reported limitations with the technique as the reference lifetime becomes long compared to the channel width or when the fluorescence lifetime of the specimen decay being fitted is shorter than the reference lifetime. We have confirmed this with our own simulations, which are presented below, and here show analytically that this is due to the discretisation of the convolution in term 2 of Eq. 2(b). These errors become greater when the temporal sampling interval is not much smaller than the combined lifetime, *τ*_*i*_′ and when *τ*_*R*_ is longer than all values of *τ*_*i*_.

For TCSPC data acquisition, photon arrival times are recorded in time-bins of a specific width and it is necessary to account for this temporal binning in a treatment of TCSPC measurements. In our model this is achieved by integrating the specimen fluorescence decay model over the width of a time bin, Δ*t*, and the DFCM for TCSPC data, when the specimen decay model is a sum of exponentials, is now as shown in Eq. 4.

4 $$ \begin{array}{c}{I}_{\mathrm{DFCM}\hbox{-} \mathrm{TCSPC}}\left({t}_j\right)={\displaystyle \sum_i\frac{a_i}{a_R}\left\{\underset{\mathrm{Term}\ 1}{\underbrace{R_{\mathrm{meas}}\left({t}_j\right)}}+\frac{1\ }{\tau_i^{\prime }}\left(\underset{\mathrm{Term}\ 2}{\underbrace{\left[A\left({t}^{\prime },{\tau}_i\right)\ast {R}_{\mathrm{meas}}\left(t^{\prime}\right)\right]\left({t}_j\right)}}\right)\right\}}\kern1.25em \left(\mathrm{a}\right)\hfill \\ {}\hfill {R}_{\mathrm{meas}}\left({t}_j\right)={\int}_{t_j}^{t_j+\varDelta t}R(t)\mathrm{d}t\kern2em \left(\mathrm{b}\right)\hfill \end{array} $$

Here, *t*_*j*_ is the *j*^th^ time-bin spanning *j*Δ*t* → (*j* + 1)Δ*t*. *R*_meas_ is formally what is measured when the signal from the reference fluorophore is acquired using a TCSPC instrument. This discretisation does not cause a problem for term 1 but it does for term 2 where an analytical exponential decay function is convolved with the discrete (measured) reference fluorophore decay that is only known at discrete time points *t*_*j*_.

To illustrate the problem that arises, we can follow the approach used for the validation of Eq. 2(b) above and assume a Delta function IRF such that the reference fluorescence profile follows a single exponential decay with time constant *τ*_R_. Because of the integral over the time bin, *R*_meas_ (and therefore term 1) is exactly *a*_*R*_*τ*_*R*_ [1-exp (−Δ*t*/*τ*_*R*_)] exp (−*t*_*j*_/*τ*_*R*_). However, the convolution in term 2 must be approximated by a discrete convolution since the reference decay has only been measured at a discrete number of time points. If we use simple zero order discretisation to perform the convolution, the model must be integrated over the width of a TCSPC bin and hence:

$$ {A}_{0\mathrm{t}\mathrm{h}\  order}\left({t}_j,\tau \right)={\int}_{t_j}^{t_j+\Delta t}A\left(t,\tau \right)\mathrm{d}t=\tau \left(1- \exp \left(-\frac{\Delta t}{\tau}\right)\right)A\left({t}_j,\tau \right) $$

and so Eq. 4 becomes:

5 $$ {I}_{\mathrm{DFCM}\hbox{-} \mathrm{TCSPC}}\left({t}_j\right)={\displaystyle \sum_i\frac{a_i}{a_R}\left\{{R}_{\mathrm{meas}}\left({t}_j\right)+\underset{\alpha }{\underbrace{\frac{\tau_i\left(1- \exp \left(-\frac{\Delta t}{\tau_i}\right)\right)}{\tau_i\hbox{'}}}}\sum_{n=0}^j{R}_{\mathrm{meas}}\left({t}_n\right) \exp \left(\frac{{t_n-t}_j}{\tau_i}\right)\right\}} $$

By then substituting in the expression for *R*_meas_ gives:

6 $$ {I}_{\mathrm{DFCM}\hbox{-} \mathrm{TCSPC}}\left({t}_j\right)={\tau}_R\left(1- \exp \left(-\frac{\Delta t}{\tau_R}\right)\right){\displaystyle \sum_i{a}_i\left\{\underset{\mathrm{Term}\ 1}{\underbrace{ \exp \left(-\frac{t_j}{\tau_R}\right)}}+\frac{\tau_i\left(1- \exp \left(-\frac{\Delta t}{\tau_i}\right)\right)}{\tau_i\hbox{'}}\underset{\mathrm{Term}\ 2}{\underbrace{\sum_{n=0}^j \exp \left(\frac{-{t}_n}{\tau_R}\right) \exp \left(\frac{t_n-{t}_j}{\tau_i}\right)}}\right\}} $$

By rearranging term 2 of Eq. 6 and noting that *t*_n_ = *n*Δ*t*, it can be seen that the term is a geometric series in exp (−Δ*t*/*τ*_i_′). Equation 7 is obtained by summing the series and simplifying:

7 $$ {I}_{\mathrm{DFCM}\hbox{-} \mathrm{TCSPC}}\left({t}_j\right)={\tau}_R\left(1- \exp \left(-\frac{\Delta t}{\tau_R}\right)\right){\displaystyle \sum_i{a}_i\left\{\underset{A}{\underbrace{ \exp \left(-\frac{t_j}{\tau_R}\right)}}+\underset{B}{\underbrace{\frac{\tau_i\left(1- \exp \left(-\frac{\Delta t}{\tau_i}\right)\right)}{{\tau_i}^{\hbox{'}}\left(1- \exp \left(-\frac{\Delta t}{{\tau_i}^{\hbox{'}}}\right)\right)}}}\left(\underset{C}{\underbrace{ \exp \left(-\frac{t_j}{\tau_i}\right)}}-\underset{D}{\underbrace{ \exp \left(-\frac{\Delta t}{{\tau_i}^{\hbox{'}}}\right)}}\underset{E}{\underbrace{ \exp \left(-\frac{t_j}{\tau_R}\right)}}\right)\right\}} $$

If the DFCM is valid as presented, we now expect Eq. 7 to simplify to the original specimen model decay function (i.e. a multi-exponential decay with amplitudes and decay constants *a*_i_ and *τ*_i_). Upon inspection of Eq. 6, it can be seen that when Δ*t*/*τ*_i_ → 0 and Δ*t*/*τ*_i_′ → 0, the numerator and denominator of factor *B* both approximate to Δ*t* so *B* tends to 1, and factor *D* also tends to 1. Hence, the exponential decay terms with lifetime *τ*_R_ (terms *A* and the product of *B*, *D*, *E*) cancel, leaving only terms with exponential decay components *τ*_i_ (corresponding to term *C*). Therefore, in the limit Δ*t* → 0, Eq. 6 does indeed simplify to the original physical decay model.

However, when Δ*t* is not negligible, terms *A* and the product of *B*, *D*, *E* do not cancel and so there is a residual reference component, i.e. there is an extra decay component with lifetime *τ*_R_ and amplitude (1 – *BD*). It should be noted, however, that if the reference has a significantly shorter lifetime than the specimen, then even if the amplitude of the residual reference component is not negligible, it will decay much faster than the terms corresponding to the physical decay model, i.e. for *τ*_R_ < <*τ*_i_ we expect the DFCM to produce reasonable results. Therefore, a more suitable way to quantify the magnitude of the residual reference components is with its photon weighted amplitude relative to the specimen decay, i.e. (1 –*BD*)τ_R_/τ_i_.

We have therefore shown analytically that the DFCM will produce erroneous results due to the discretisation of term 2 of Eq. 4, which is required to compute the convolution. If we were to have used the trapezium approximation in the discretisation of the convolution term, we would obtain a similar result, except that the residual reference component would have a smaller, negative pre-exponential factor; in this case, a similar analysis shows that the amplitude of the residual reference component is ~ $-\sum_i\Delta t/2{\tau}_i\hbox{'}$ for small Δt compared to ~$\sum_i\Delta t/{\tau}_i\hbox{'}$ for zero order discretisation. In fact, there will always be some mismatch between the terms 1 and 2 of Eq. 4 for any method of interpolation and so there will always be a residual reference component when computing the DFCM in this fashion. We note that any more complex method of interpolation will impact the computation time required to fit the decay curves, especially when fitting multi-exponential decay models over large data sets. Therefore, instead of improving the interpolation, we propose to introduce a correction factor to compensate for the residual reference component.

Since the deviation in the DFCM from the physical decay is quantified by factors *B* and *D* in Eq. 7, we can apply a correction factor to the convolution term in Eq. 5, which is given by:

8 $$ \frac{1}{BD}=\frac{\tau_i\hbox{'}\left( \exp \left(\Delta t/{\tau}_i\hbox{'}\right)-1\right)}{\tau_i\left(1- \exp \left(-\Delta t/{\tau}_i\right)\right)} $$

By applying our correction factor (the RHS of Eq. 8) to *α* in Eq. 5 and then simplifying the result, we obtain Eq. 9(a).

9 $$ \begin{array}{l}{I}_{\mathrm{CORR}}\left({t}_j\right)={\displaystyle \sum_i}{a}_i^{\prime}\left\{{R}_{\mathrm{meas}}\left({t}_j\right)+\left( \exp \left(\frac{\varDelta t}{\tau_i^{\prime }}\right)-1\right){\displaystyle \sum_{n=0}^j} \exp \left(\frac{t_n-{t}_j}{\tau_i}\right){R}_{\mathrm{meas}}\left({t}_n\right)\right\}\kern2em (a)\hfill \\ {}\hfill {a}_i=\frac{\tau_R\left( \exp \left(\raisebox{1ex}{$\varDelta t$}\!\left/ \!\raisebox{-1ex}{${\tau}_R$}\right.\right) - 1\right)}{\tau_i\left( \exp \left(\raisebox{1ex}{$\varDelta t$}\!\left/ \!\raisebox{-1ex}{${\tau}_i$}\right.\right) - 1\right)}\ {a}_i^{\prime}\kern1em (b)\hfill \\ {}\hfill {Res}_{\mathrm{CORR}}\left({t}_j\right)=\frac{I_{\mathrm{CORR}}\left({t}_j\right)-{I}_{\mathrm{meas}}\left({t}_j\right)}{\sqrt{I_{\mathrm{CORR}}\left({t}_j\right)+{\left({\displaystyle {\sum}_i}\ {a}_i^{\prime}\right)}^2{R}_{\mathrm{meas}}\left({t}_j\right)}}(c)\hfill \end{array} $$

The application of the correction factor means that we also need to correct the decay amplitude contributions, which is shown in Eq. 9(b). When this corrected model is evaluated for a Delta function IRF as before, a multi-exponential decay is obtained with lifetime components *τ*_i_ and with no unwanted term with decay constant *τ*_*R*_.

For completeness, the residuals function used for fitting is shown in Eq. 9(c), where *I*_meas_(*t*) is the measured fluorescence decay profile of the specimen under inspection. As is normal for least squares fitting, the residuals function is weighted by the expected noise at each data point. In this case, the weighting is unchanged from that used in conventional DFCM since the correction factor has been applied to the convolution term which has a negligible variance compared to the measured reference decay [17].

In this section, we have shown that discretisation of the conventional DCFM model by the width of a time-bin leads to an unwanted term in the final equation and that this can be overcome through the introduction of a correction to the DFCM model. The discretisation of the model over a time-bin is equivalent to assuming an IRF with a top-hat profile and therefore an experimentally acquired IRF can be modelled by summing the results obtained from a series of top-hat segments of appropriate amplitudes, i.e. zero-order interpolation of the IRF. In the following sections, we will show that by evaluating the corrected DFCM (Eq. 9) in the case of data simulated using a realistic IRF and for given specimen decay parameters, the resulting curve correctly matches the specimen decay model. We then validate that this approach can be used in decay fitting by using Monte-Carlo simulations to show that, when fitting using the corrected DFCM, the correct decay parameters are retrieved. In these simulations, we validate our use of zero-order interpolation by generating the IRF and the data at a high temporal resolution and then binning it to a lower temporal resolution before curve fitting. In addition, we show that the corrected DFCM yields comparable results to impulse convolution over a wide range of lifetimes and offers a substantial improvement over the conventional DFCM. We also demonstrate the use of DFCM in fitting experimentally acquired data.

## Methods

### Non-Linear Least Squares Fitting

Non-linear least squares fitting of simulated or experimental TCSPC data was employed using the Levenberg-Marquadt minimisation algorithm in MATLAB® (The Mathworks, Inc., Massachusetts). Three types of fitting model were implemented: impulse convolution, conventional DFCM and corrected DFCM. Impulse convolution is the commonly used method of forward convolution of the model decay function with a directly measured IRF, e.g. from a scattering specimen, which we will term IRF_meas_. In this case, we found that it was necessary to use the trapezium approximation to evaluate the convolution. This compensates for the sub-resolution temporal variation in an IRF acquired from a scatterer (i.e. the IRF is not constant within a time-bin measurement). We found that the trapezium approximation was not necessary when using the corrected DFCM since the sub-resolution temporal variation in the reference decay becomes negligible when this is much longer than the width of a time-bin. For the same reason, the trapezium approximation should not be necessary for the conventional DFCM. However, for a fair comparison with the impulse convolution method using trapezium integration, we have implemented the trapezium approximation in the conventional DFCM. The resulting equations are shown in Eq. 10: (a) & (b) show the model decay and residuals functions for impulse convolution and (c) & (d) show these functions for the conventional DFCM. The terms shown in Eq. 10(e) arise due to the trapezium approximation and can be derived by linear interpolation of the IRF function followed by convolution with the exponential decay function. The corresponding functions for the corrected DFCM are as shown in Eq. 9. In these equations, the terms and conventions are as stated above.

10 $$ \begin{array}{l}{I}_{\mathrm{MOD}}\left({t}_j\right)={\displaystyle \sum_i}{a}_i{\displaystyle \sum_{n=0}^j}\frac{C_1\ {IRF}_{\mathrm{meas}}\left({t}_n\right)+{C}_2{\ IRF}_{\mathrm{meas}}\left({t}_{n+1}\right)}{2}\  \exp \left(\frac{t_n-{t}_j}{\tau_i}\right)\kern2.25em (a)\hfill \\ {}{Res}_{\mathrm{MOD}}\left({t}_j\right)=\frac{I_{\mathrm{MOD}}\left({t}_j\right)-{I}_{\mathrm{meas}}\left({t}_j\right)}{\sqrt{I_{\mathrm{MOD}}\left({t}_j\right)}}\kern2.5em (b)\hfill \\ {}\hfill {I}_{\mathrm{DFCM}\hbox{-} \mathrm{TCPSC}}\left({t}_j\right)={\displaystyle \sum_i}{a}_i\left\{{R}_{\mathrm{meas}}\left({t}_j\right)+\frac{1}{\tau_i^{\prime }}{\displaystyle \sum_{n=0}^j}\frac{C_1\ {\mathrm{R}}_{\mathrm{meas}}\left({t}_n\right)+{C}_2\ {R}_{\mathrm{meas}}\left({t}_{n+1}\right)}{2}\  \exp \left(\frac{t_n - {t}_j}{\tau_i}\right)\right\}\kern2.5em (c)\hfill \\ {}\hfill {Res}_{\mathrm{DFCM}\hbox{-} \mathrm{TCSPC}}\left({t}_j\right)=\frac{I_{\mathrm{DFCM}}\left({t}_j\right)-{I}_{\mathrm{meas}}\left({t}_j\right)}{\sqrt{I_{\mathrm{DFCM}}\left({t}_j\right)+{\left({\displaystyle {\sum}_i}\ {a}_i^{\prime}\right)}^2{R}_{\mathrm{meas}}\left({t}_j\right)}}\kern2em (d)\hfill \\ {}\hfill \begin{array}{ll}{C}_1={\tau}_i\left\{\frac{\tau_i}{\varDelta t}\left( \exp \left(\raisebox{1ex}{$\varDelta t$}\!\left/ \!\raisebox{-1ex}{${\tau}_i$}\right.\right)-1\right)-1\right\}\hfill & \kern1em {C}_2={\tau}_i\left\{ \exp \left(\raisebox{1ex}{$\varDelta t$}\!\left/ \!\raisebox{-1ex}{${\tau}_i$}\right.\right) - \frac{\tau_i}{\varDelta t}\left( \exp \left(\raisebox{1ex}{$\varDelta t$}\!\left/ \!\raisebox{-1ex}{${\tau}_i$}\right.\right)-1\right)\right\}\hfill \end{array}\kern2em (e)\hfill \end{array} $$

In order to reduce bias commonly encountered with non-linear least squares fitting at low photon counts, data points with fewer than 10 counts were ignored. It should be noted that we are not performing our simulations in a low signal level regime and therefore precise determination of a lower threshold was not required. If operating in a low signal level regime, the maximum likelihood method for curve fitting may be more appropriate.

### Simulated Data

Simulated data was generated using MATLAB®. An IRF was generated by summing four Gaussian curves at a temporal resolution of 8192 time bins over a range of 12.5 ns. The Gaussian parameters were chosen such that the final curve resembled an experimentally acquired IRF from a Hamamatsu H7422P-40 and a B&H SPC-830 TCSPC card with a FWHM of 500 ps. The parameters (amplitude, temporal offset and standard deviation) for the four Gaussians were: 100, 2.44, 0.15; 1, 2.44, 0.3; 1.5, 3.66, 0.15 and 1.2, 4.54, 0.15 respectively. The resultant IRF will be referred to as the “realistic IRF”. Single or double exponential decays were generated with 8192 time bins over a range of 12.5 ns. The exponential decay curves were then convolved with the realistic IRF (with zero-order interpolation) and the resulting curves were then binned down to 256 time bins. Generating the data at a high temporal resolution and then re-binning it in this fashion accounts for the fact that the actual impulse response does vary (slowly) within an experimental time bin. We are thus able to validate our choice of zero-order discretisation of the convolution when fitting using the modified DFCM.

When performing curve fitting, the simulated decay curves were scaled to a signal level of 10^6^ counts in total and Poisson noise was added. When performing DFCM, reference decays with single exponential lifetimes of 1.0 ns and 2.0 ns and 3 × 10^6^ total counts were generated in the same fashion as the decay curves including the addition of noise. When performing conventional impulse convolution, the high temporal resolution IRF was binned down to 256 time bins, scaled in amplitude such that the count at the peak was 5 × 10^5^ and then Poisson noise was added. This count level was chosen since it is just below the maximum count obtainable (16-bit) by some of the hardware typically used in fluorescence lifetime measurements (e.g. B&H TCSPC cards).

For the Monte-Carlo simulations, each decay curve was generated 16 times with different noise and fitted using the three model decay functions (i.e. the same set of 16 noisy decay curves were fitted using impulse convolution, the conventional DFCM and the corrected DCFM). The mean and sample standard deviation of each fitted parameter was then calculated for each model. The corrected DFCM was tested using single and double exponential decays. Single exponential lifetimes were chosen to be between 0.2 ns and 2.0 ns. For double exponential decays, the short component was fixed at 0.2 ns, the long component ranged from 0.4 ns to 2.0 ns and the photon-weighted amplitudes of the two components were equal (i.e. *a*_1_*τ*_1_ = *a*_2_*τ*_2_). A signal level of 10^6^ was simulated for both the double and single exponential decays.

### Experimental Data Acquisition

Experimental data was acquired using a custom built TCSPC based fluorescence lifetime spectrometer described elsewhere [24]. Briefly, a supercontinuum laser source (SC400, Fianium) was directed into a prism based spectral selection unit to tune the excitation wavelength. Excitation light was then passed through a Glan-Taylor polarising cube before being directed onto a Cuvette containing the analyte. Fluorescence from the specimen was collected at right angles to the excitation beam and imaged through an emission filter (FF01-585/40–25, Semrock) and polarizer onto the input slit of a monochromator (CM110, CVI inc.) and detected by a photomultiplier tube (PMC-100, B&H, GmBH). TCSPC measurements were performed using a B&H SPC-730 card. For the measurements presented in this paper, an excitation wavelength of 525 nm at 20 MHz was used. The selected emission fluorescence was centred at 580 nm with a bandwidth of 10 nm. The TCSPC resolution was set to 1024 bins, equal to a bin width of approximately 50 ps. To perform impulse convolution, the IRF of the system was acquired with a sample of Ludox (LUDOX® TM-40 colloidal silica, Sigma Aldrich) as a specimen, which entailed removing the emission filter and centring the monochromator at the excitation wavelength. All measurements were recorded with the emission polariser at the magic angle to the excitation polarisation to ensure that the polarisation of the specimen did not affect the fluorescence decay [1].

The experimentally acquired data has a significant and unavoidable d.c. offset due to after-pulsing of the PMT. To compensate for this, the offset was estimated using a 5 ns portion of the data prior to the arrival of the excitation pulse. This method of offset estimation is valid since the period of 50 ns between excitation pulses was sufficient to assume that the fluorescence from any of the specimens had decayed completely. The measured offset of the decay curve under inspection was included as a fixed parameter in the fitting model. For the measured impulse IRF and reference decays, the offset was subtracted from the measured profiles before use in the fitting routine. The long period between excitation pulses also meant that there was no requirement to compensate for incomplete decays in the model.

The fluorophores chosen and their reported fluorescence lifetimes [20, 25] were: Rhodamine 6G in water (4.08 ns), Rhodamine B in water (1.68 ns–1.74 ns), Erythrosin B in methanol (0.47 ns) and Erythrosin B in water (0.089 ns). Rhodamine B was chosen as a reference fluorophore with its lifetime fixed to the value we obtained by fitting with impulse convolution (1.656 ns) using a scattered excitation light measurement. Data was acquired from each fluorophore three times and NLLSQ fitting with the three models (each using a single exponential decay model) was performed as described above.

## Results

Figure 1 shows the conventional and corrected DFCM computed with fixed parameters in comparison to the desired noiseless monoexponential specimen decay model. In Fig. 1a, the corrected DCFM was obtained using Eq. 9 and with the reference decay (R_meas_) set to a noiseless pure monoexponential decay with lifetime τ_Ref_. It can be seen that in the very early part of the decay, the conventional DFCM matches the physical decay model. However, the conventional DFCM deviates from the physical model in the later part of the decay. For the conventional DCFM calculated with zero order (simple) interpolation, the conventional DFCM runs parallel to the reference decay curve as is expected from the theory section, see e.g. Eq. 7. When using trapezium interpolation the deviation starts later and is in the opposite direction. In this case, the model decay goes negative for a while before rising to zero (not shown on the log scale). However, it can be seen that the corrected DFCM model matches the specimen decay curve. The same features can be seen when the noiseless realistic IRF is used, see Fig. 1b. The corrected DFCM was calculated using zero-order interpolation but, as can be seen from Fig. 1a, b, it yields the desired specimen decay, which indicates that this approach is valid.

**Fig. 1:**
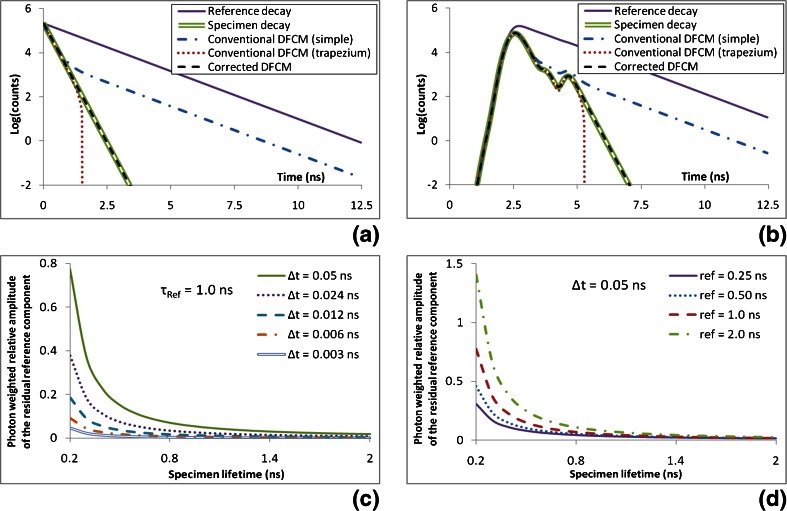
Showing the unwanted residual reference decay component in conventional DFCM. **a** and **b** show the conventional and corrected DFCM computed for a specimen with 0.2 ns fluorescence decay time, a reference with 1.0 ns fluorescence decay time and a time bin width of 0.05 ns. The two types of conventional DFCM, simple and trapezium, use zero or first order interpolation respectively to evaluate the convolution in Eq. 4. The corrected DFCM is computed using Eq. 9 which, as described in the text, uses zero order interpolation. The reference and specimen decay curves were generated using an ideal IRF (i.e. a Delta function at time = 0) in (**a**) and the realistic IRF in (**b**). Noise was not added to any of the curves shown in this figure. **c** and **d** show the photon weighted amplitude of the reference decay component relative to the specimen decay. This is plotted as a function of the specimen lifetime for a range of time bin widths at a fixed reference lifetime in (**c**) and for a range of reference lifetimes at a fixed time bin width in (**d**)

As expected, the photon weighted amplitude of the (unwanted) residual reference decay component relative to the specimen decay increases as the bin width increases and as the specimen lifetime decreases (Fig. 1c) or the reference lifetimes increases (Fig. 1d). It can be seen that even for the smallest bin width used (*Δt* = 0.003 ns) and the reference and specimen lifetimes shown in Fig. 1c, the relative magnitude of the unwanted residual reference component is still a few percent of that of the specimen. When using a bin width equivalent to 256 time bins over 12.5 ns (Δt = 0.05 ns), it can be seen that even when using a reference fluorophore with a lifetime of 0.25 ns, the relative magnitude of the residual reference component is 30 % for specimen fluorescence lifetimes of 0.2 ns (Fig. 1d).

Figure 2 shows the fitting of individual simulated curves using the three different models together with the resulting normalised residuals. It can be seen that when fitting a 0.2 ns single exponential decay (Fig. 2c), the conventional DFCM fails to produce a good fit to the data; there is a systematic deviation from zero in the residuals around the rising edge of the curve. Figure 2e shows that the curve fitted using the corrected DFCM yields a better fit to the data with residuals randomly distributed around zero. The corrected DFCM retrieved the correct lifetime, whereas the conventional DFCM returned an overestimate.

**Fig. 2:**
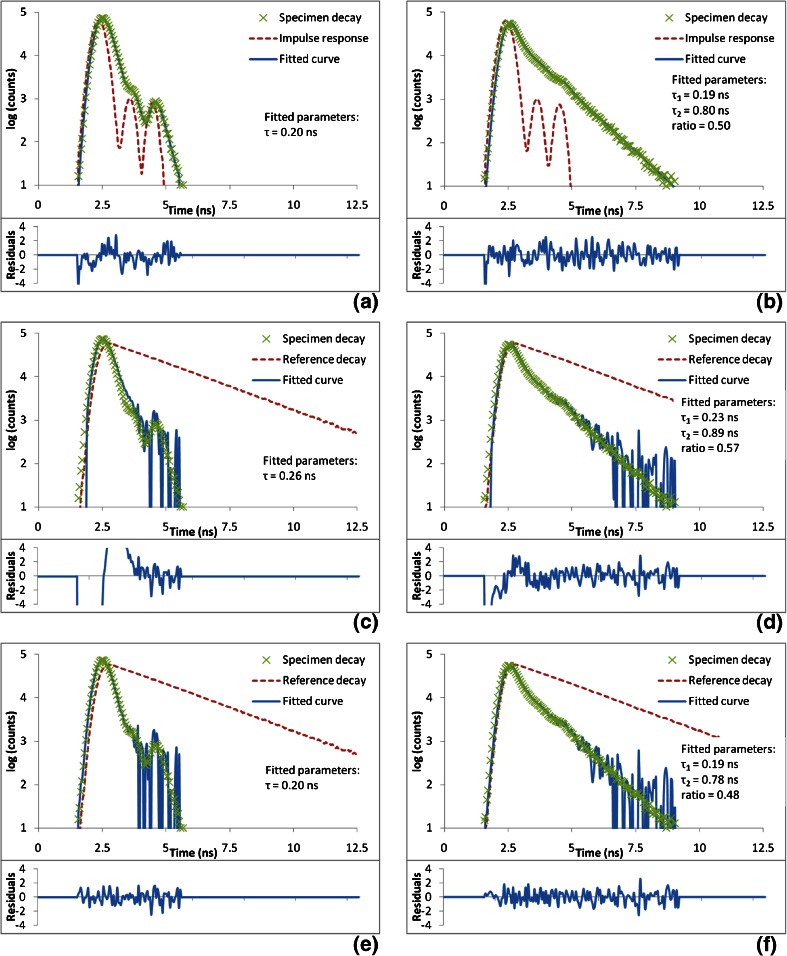
Shows the performance of the different fitting models on individual simulated decay curves. The left hand column shows data simulated with a single exponential decay of 0.2 ns. The same data is used in (**a**), (**c**) and (**e**) and the same reference decay is used in (**c**) and (**e**). The right hand column shows data simulated with a double exponential decay with lifetimes of 0.2 ns and 0.8 ns and a photon weighted amplitude ratio of 0.5. The same data is used in (**b**), (**d**) and (**f**) and the same reference decay is used in (**d**) and (**f**). The reference was simulated with a decay time of 2.0 ns. The realistic IRF (the *red curve* in (**a**) and (**b**)) was used to generate the specimen and reference decays. The total number of simulated photons was 10^6^ in all specimen decay curves and 3 × 10^6^ in all reference decay curves. The *top row* shows the data and curves fitted with the ideal method of impulse convolution. The *middle row* shows the data fitted using the conventional DCFM. The *bottom row* shows the data fitted with the corrected DCFM. The *upper plot* of each panel shows the fitted curves and the *lower plot* of each panel shows the weighted residuals

When fitting a double exponential decay, see Fig. 2d, the conventional DFCM model has produced a good fit in the latter portion of the curve, but at the rising edge of the curve, there is a deviation from zero in the residuals. The fitted parameters do not match the simulated values or those returned by impulse convolution. Figure 2f shows that the corrected reference model returns a good fit and that the fitted parameters are much closer to the simulated values than those returned by the conventional DFCM.

Experimentally acquired data obtained from erythrosine B fitted with the conventional and corrected DFCM methods (Fig. 3) shows the same behaviour as the simulated data. It can be seen that when the specimen has a lifetime that is shorter than that of the reference, there is a systematic deviation from zero in the residuals around the rising edge of the curve. The fitted lifetime (0.16 ns) is not in agreement with the value reported by Boens et al. (0.089 ± 0.003 ns) [20]. However, when the same data set was fitted with the corrected DFCM, the residuals behave as expected and the fitted lifetime value (0.09 ns) is in agreement with that reported by Boens et al. and with that obtained by fitting with impulse convolution (0.09 ns - data presented in Table 1).

**Fig. 3:**
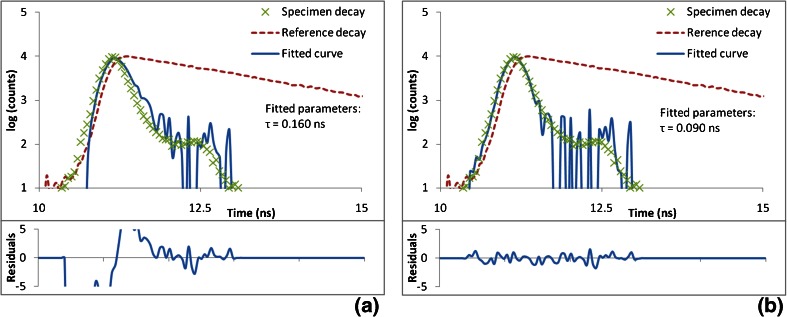
Shows fits obtained with **a** the conventional DFCM and **b** the corrected DFCM to an experimentally acquired decay from erythrosine B in water. The lifetime of the reference fluorophore, Rhodamine B in water, was measured to be 1.656 ns, which was obtained by fitting the reference decay using the impulse convolution model with a scatterer IRF measurement acquired using Ludox. The bin width used in the data acquisition was 0.0488 ns

**Table 1:** Shows the performance of the three fitting techniques on experimentally acquired data. The reported lifetime values are from [20] (†) and [25] (‡). The fitted lifetimes are the mean ± standard deviation of three measurements. When using the two DFCM methods, the references used were Rhodamine B in water and Rhodamine 6G in water. Their lifetimes were fixed to the value obtained by fitting with impulse convolution (1.656 ns for Rhodamine B and 4.010 ns for Rhodamine 6G)

Type	Fluorophore (solvent)	Reported lifetime (ns)	Fitted lifetime (ns)			
			Impulse convolution	Reference	Conventional DFCM	Corrected DFCM
Specimen	Erythrosin B (water)	0.089 ± 0.003 †	0.089 ± 0.001	Rhodamine 6G	0.189 ± 0.001	0.087 ± 0.001
				Rhodamine B	0.158 ± 0.003	0.089 ± 0.002
Specimen	Erythrosin B (methanol)	0.47 ± 0.02 †	0.469 ± 0.001	Rhodamine 6G	0.483 ± 0.004	0.460 ± 0.001
				Rhodamine B	0.474 ± 0.001	0.473 ± 0.001
Reference	Rhodamine 6G (water)	4.08 ‡	4.01 ± 0.01	–	–	–
Reference	Rhodamine B (water)	1.74 ± 0.02 † 1.68 ‡	1.656 ± 0.001	–	–	–

Figure 4 shows the results of the Monte-Carlo simulations. In these simulations, fluorescence decay values were based on those of autofluorescence molecules found in tissue. The most difficult decay curves to fit are those which contain components with short, closely spaced decay times. Therefore, we have chosen exponential decays with a short component of 0.2 ns, corresponding to melanin [26] and a long component increasing from 0.4 ns, similar to that of free NAD(P)H [27], up to 1.5 ns. We have also simulated single exponential decays using the same range of lifetimes. We have chosen reference decay times of 1 ns and 2 ns which are typical of those recommended for use as fluorescence lifetime standards [20].

**Fig. 4:**
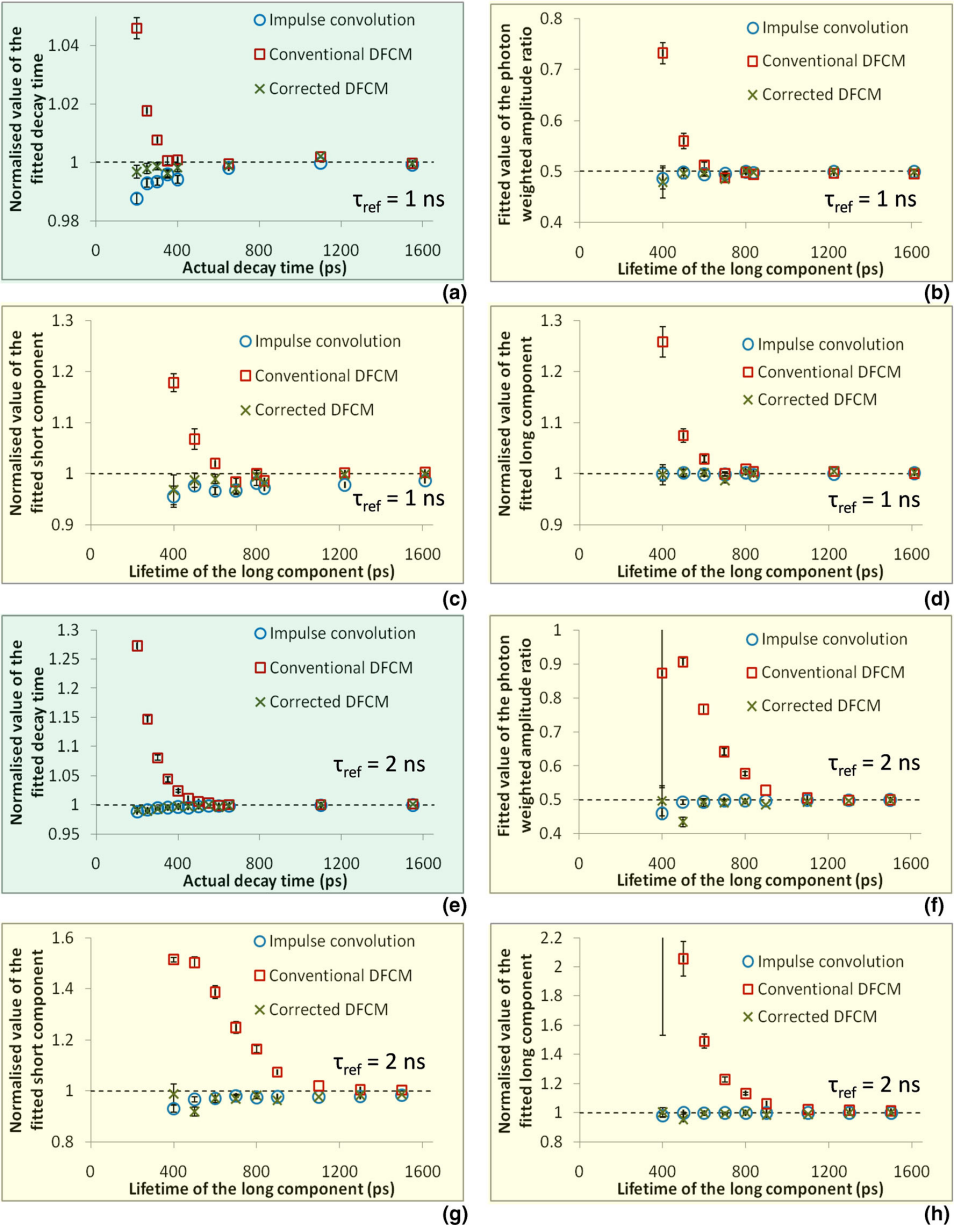
Shows results of Monte-Carlo simulations of exponential decay curves fitted with the three different models and using a simulated reference decay with lifetimes of 1 ns (**a**–**d**) and 2 ns (**e**–**h**). The single exponential decay curves (panels **a** & **e**, *shaded green*) were simulated with lifetimes varying from 0.2 ns to 1.5 ns. Double exponential decay curves (panels **b**–**d** & **f**–**h**, *shaded yellow*) were simulated with the short component fixed at 0.2 ns, the long component varying from 0.40 to 1.5 ns and equal photon weighted amplitudes. In the fitted lifetime plots (panels **a**, **c**, **d**, **e**, **g**, and, **h**), the mean ± standard deviation were divided by the simulated lifetime so that a value of unity corresponds to an exact fit. In the amplitude ratio plots (panels **b** and **f**), a value of 0.5 corresponds to an exact fit

For the single exponential simulated decays (shown in Fig. 4a, e), it can be seen that the conventional DFCM returns acceptable lifetimes for long specimen decays. However, for short specimen decay times, there is a systematic overestimate of the fitted lifetime that is much larger than the error bars. In contrast, the corrected DFCM has returned lifetimes which are comparable in accuracy to those returned by impulse convolution.

For the double exponential simulated decays (Fig. 4b–d and f–h), similar results are obtained; as the long component of the double exponential gets shorter, a systematic deviation larger than the error bars emerges between the fitted and the simulated parameters. When fitting with a reference lifetime of 2 ns, the systematic error in all of the returned parameters is less than 5 % only when the long lifetime is greater than 1 ns. However, the corrected DFCM has fitted parameters that are comparable to the impulse convolution method across the whole range of reference and specimen lifetimes. In both cases of single and double exponential decays, the magnitude of the error and the range of affected lifetimes is larger when the reference decay time is longer. It can also be seen that the range of affected lifetimes is larger when fitting the double exponential data.

When we apply the conventional and corrected DFCM to experimentally acquired data, our results, presented in Table 1, show a similar trend to our Monte Carlo simulations. It can be seen that the conventional DFCM has performed well for long lifetimes, but poorly for short ones. However, the corrected DFCM performs as well as impulse convolution for all the fluorophores tested.

## Discussion

The use of a reference fluorophore with known characteristics in the temporal calibration of fluorescence lifetime instrumentation is a well-established technique. The measured fluorescence decay profile can be used in a variety of ways, but the most common technique is to use the Delta function convolution method (DFCM) with non-linear least squares fitting. Although this approach has been described as mathematically rigorous and has sometimes been shown to provide excellent results [10, 17, 21, 22], it has also been found to be unreliable under certain conditions. The unreliability has been noted to be associated with reference lifetimes longer than the temporal sampling interval, the IRF and the specimen decays [10, 14, 21--23]. However, to date there has been no report of a systematic study into the conditions under which the DFCM fails and no conclusive evidence of its cause. We have shown for the first time, to the best of our knowledge, that the cause of the error is a mismatch between the two terms of the modified model function which arises because of the unavoidable discretisation error associated with the numerical convolution in one of the terms of the modified model function. Our analysis shows that the error is quantifiable for multi-exponential decays and we have therefore been able to suggest a correction to the DFCM.

Our theoretical analysis of the conventional DFCM model decay function has shown that it has a residual reference component in addition to the physical decay component(s), i.e. for given reference and specimen exponential decay parameters, the computed conventional DFCM model deviates from the specimen model by the presence of a term that matches the reference decay. It is this residual reference component that leads to the errors when using the conventional DFCM under certain conditions. In this study, we have chosen to simulate the short fluorescence lifetimes similar to those associated with tissue and cellular autofluorescence (melanin [28] and NAD(P)H [27]) which are commonly encountered experimentally, for example in [29--32]. We have chosen reference decay times similar to many of the available reference fluorophores presented by Boens et al. in their comprehensive review of fluorescence standards for time resolved measurements [20].

In our Monte-Carlo simulations of single exponential fluorescence lifetime data, we have shown that for long fluorescence lifetimes, the three model functions perform as well as each other. However, as the specimen decay gets shorter, the conventional DFCM returns an overestimate of the true value. This behaviour is explained in the following way: when the specimen fluorescence lifetime is shorter than that of the reference, the latter portion of the conventional DFCM model curve (computed with trapezium integration) is dominated by the residual reference component which lies beneath the true curve as shown in Fig. 1a, b and the fitting routine compensates for this by overestimating the fitted lifetime. This effect becomes smaller as the specimen decay time gets longer and lies within the noise of our Monte Carlo simulations. This may explain why many studies have not reported any systematic errors with the conventional DFCM when using reference decay times that are shorter than the specimen decay times.

In our simulations, we have used count levels for the specimen and references decay curves (10^6^ and 3 × 10^6^ photons per decay respectively), which can be realistically obtained in single point measurements of tissue autofluorescence in a reasonable period of time. For example: Coda et al. achieved a count rate of 6 × 10^5^ s^−1^ from colonic autofluorescence [33] (based on an average of 2 × 10^5^ counts in each of 16 spectral channels acquired in 5 s); Skala et al. achieved count rates of 5 × 10^5^ s^−1^ with multiphoton excitation in a hamster cheek pouch model and in live cells [31] and in our experience with skin and heart tissue autofluorescence, similar count rates are easily obtained (unpublished data). Therefore we have shown that the results of the corrected DCFM can in practice be as accurate as those obtained by impulse convolution across a wide range of lifetimes. We have also confirmed this in our tests on experimental data. Our simulations show that the magnitude of the overestimate and the range of affected lifetimes increase as the reference decay time increases. We have also shown that the range of lifetimes affected is larger for multi-exponential decay curves. We emphasise that this range overlaps with decay times found in autofluorescence data [29--32] and therefore recommend that our correction be implemented if the DFCM is to be reliably applied in, for example, the growing field of tissue FLIM.

One limitation of the correction presented in this work is that it is only applicable to cases where the specimen model is a multi-exponential decay and we have only evaluated the correction factor for TCSPC style data, i.e. time bins with constant spacing equal to the bin width. However, these are not significant drawbacks since firstly, the majority of fluorescence lifetime experiments utilise a (multi‑) exponential decay model in their analysis. Secondly, if a different model decay function is required or a method other than TCSPC is used to acquire the data, a correction factor can be calculated using the analysis technique we have outlined. The correction factor will be expressed as summation which in some cases may be simplified in a similar way to that shown above (the summation of a geometric progression). When this is not possible, the correction factor can evaluated numerically.

One further phenomenon we have observed but not presented, is that DCFM fitting routine begins to fail when the number of counts in the measured reference decay is similar to, or smaller than the specimen counts. We have found that acceptable results are obtained once the measured reference fluorescence profile has about three times (or greater) the number of counts as in the specimen decay curve. We believe that this particular constraint can be easily met during real-life data acquisition.

In conclusion, we have shown analytically that the conventional DFCM model used in fitting fluorescence decay curves is subject to discretisation errors that can degrade accuracy of fitted model parameters. The errors become particularly significant when the following two conditions are simultaneously met: firstly, when the fluorescence lifetime of the reference fluorophore is longer than that of all individual components of the specimen under inspection and secondly, when the temporal sampling interval is not much smaller than the combined lifetime (τ_i_′ = 1/(1/τ_R_ – 1/τ), where τ_R_ and τ_i_ are the reference and specimen decay times respectively). We have also proposed a corrected DFCM model decay function. We have shown, using both simulated and experimental data, that this is a robust model yielding results equivalent to those using impulse convolution. Since the model is a reference decay technique that can utilise a wide range of fluorophores, it can greatly reduce the effects of any spectral dependencies in the instrument response function and allows for the straight forward acquisition of a temporal calibration measurement for a wide-range of fluorescence lifetime instrumentation.

## References

[1] Lakowicz J. R. (2006). Principles of fluorescence spectroscopy.

[2] Berezin MY, Achilefu S (2010) Fluorescence lifetime measurements and biological imaging. Chem Rev 110; 2641–2684. ISSN 0009-2665 10.1021/cr900343z PMC2924670 20356094

[3] Wahl P., Auchet J. C., Donzel B. (1974). Wavelength dependence of response of a pulse fluorometer using single photoelectron counting method. Rev Sci Instrum.

[4] Krahl R, Bülter A, Koberling F (2005) Performance of the micro photon devices PDM 50CT SPAD detector with PicoQuant TCSPC systems. [S.l.]

[5] Talbot C. B. (2011). Application of ultrafast gold luminescence to measuring the instrument response function for multispectral multiphoton fluorescence lifetime imaging. Opt Express.

[6] Luchowski R. (2009). Forster resonance energy transfer (FRET)-based picosecond lifetime reference for instrument response evaluation. Meas Sci Technol.

[7] Luchowski R (2009). Instrument response standard in time-resolved fluorescence. Rev Sci Instrum.

[8] Szabelski M. (2009). Evaluation of instrument response functions for lifetime imaging detectors using quenched Rose Bengal solutions. Chem Phys Lett.

[9] Luchowski R. (2010). Fluorescence instrument response standards in two-photon time-resolved spectroscopy. Appl Spectrosc.

[10] Kolber Z. S., Barkley M. D. (1986). Comparison of approaches to the instrumental response function in fluorescence decay measurements. Anal Biochem.

[11] Wagnieres G. A., Star W. M., Wilson B. C. (1998). In vivo fluorescence spectroscopy and imaging for oncological applications. Photochem Photobiol.

[12] Chudakov D. M. (2010). Fluorescent proteins and their applications in imaging living cells and tissues. Physiol Rev.

[13] Brochon J. C. (1976). Pulse fluorimetry study of beef-liver glutamate dehydrogenase-reduced nicotinamide adenine-dinucleotide phosphate complexes. Biochemistry.

[14] Gauduchon P., Wahl P. (1978). Pulsefluorimetry of tyrosyl peptides. Biophys Chem.

[15] Ricka J. (1981). Evaluation of nanosecond pulse-fluorometry measurements - no need for the excitation-function. Rev Sci Instrum.

[16] Libertini L. J., Small E. W. (1984). F/f deconvolution of fluorescence decay data. Anal Biochem.

[17] Zuker M. (1985). Delta-function convolution method (dfcm) for fluorescence decay experiments. Rev Sci Instrum.

[18] Carraway E. R. (1985). Luminescence lifetime measurements - elimination of phototube time shifts with the phase plane method. Anal Chem.

[19] Vecer J. (1993). Reconvolution analysis in time-resolved fluorescence experiments - an alternative approach - reference-to-excitation-to-fluorescence reconvolution. Rev Sci Instrum.

[20] Boens N. (2007). Fluorescence lifetime standards for time and frequency domain fluorescence spectroscopy. Anal Chem.

[21] Van Den Zegel M. (1986). Possibilities and limitations of the time-correlated single photon-counting technique - a comparative-study of correction methods for the wavelength dependence of the instrument response function. Chem Phys.

[22] Boens N. (1988). On the use and the performance of the delta-function convolution method for the estimation of fluorescence decay parameters. Chem Phys.

[23] Löfroth J. E. (1985). Deconvolution of single photon-counting data with a reference method and global analysis. Eur Biophys J Biophys Lett.

[24] Manning H. B. (2008). A compact, multidimensional spectrofluorometer exploiting supercontinuum generation. J Biophotonics.

[25] ISS Lifetime Data of Selected Fluorophores (Online). Available: http://www.iss.com/resources/reference/data_tables/LifetimeDataFluorophores.html. Accessed June 2014

[26] Teuchner K. (2000). Fluorescence studies of melanin by stepwise two-photon femtosecond laser excitation. J Fluoresc.

[27] Wakita M., Nishimura G., Tamura M. (1995). Some characteristics of the fluorescence lifetime of reduced pyridine-nucleotides in isolated-mitochondria, isolated hepatocytes, and perfused-rat-liver in-situ. J Biochem.

[28] Teuchner K. (1999). Femtosecond two-photon excited fluorescence of melanin. Photochem Photobiol.

[29] Dimitrow E. (2009). Spectral fluorescence lifetime detection and selective melanin imaging by multiphoton laser tomography for melanoma diagnosis. Exp Dermatol.

[30] Patalay R. (2012). Multiphoton multispectral fluorescence lifetime tomography for the evaluation of basal cell carcinomas. PLoS One.

[31] Skala M. C. (2007). In vivo multiphoton microscopy of NADH and FAD redox states, fluorescence lifetimes, and cellular morphology in precancerous epithelia. Proc Natl Acad Sci U S A.

[32] Giorgi V. D. (2009). Combined non-linear laser imaging (two-photon excitation fluorescence microscopy, fluorescence lifetime imaging microscopy, multispectral multiphoton microscopy) in cutaneous tumours: first experiences. J Eur Acad Dermatol Venereol.

[33] Coda S. (2014). Fluorescence lifetime spectroscopy of tissue autofluorescence in normal and diseased colon measured ex vivo using a fiber-optic probe. Biomed Optic Express.

